# Chronic exposure to lipopolysaccharides as an *in vitro* model to simulate the impaired odontogenic potential of dental pulp cells under pulpitis conditions

**DOI:** 10.1590/1678-7757-2023-0032

**Published:** 2023-07-24

**Authors:** Igor Paulino MENDES SOARES, Caroline ANSELMI, Maria Luiza Barucci Araujo PIRES, Rafael Antonio de Oliveira RIBEIRO, Maria Luísa LEITE, Diana Gabriela SOARES, Carlos Alberto DE SOUZA COSTA, Josimeri HEBLING

**Affiliations:** 1 Universidade Estadual Paulista Faculdade de Odontologia de Araraquara Departamento de Materiais Odontológicos e Prótese Araraquara SP Brasil Universidade Estadual Paulista - UNESP, Faculdade de Odontologia de Araraquara, Departamento de Materiais Odontológicos e Prótese, Araraquara, SP, Brasil.; 2 Universidade Estadual Paulista Faculdade de Odontologia de Araraquara Departamento de Morfologia e Clínica Infantil Araraquara SP Brasil Universidade Estadual Paulista - UNESP, Faculdade de Odontologia de Araraquara, Departamento de Morfologia e Clínica Infantil, Araraquara, SP, Brasil.; 3 Department of Oral Health Sciences The University of British Columbia School of Dentistry Vancouver Canada Department of Oral Health Sciences, The University of British Columbia, School of Dentistry, Vancouver, Canada.; 4 Universidade de São Paulo Faculdade de Odontologia de Bauru Departamento de Dentística, Endodontia e Materiais Odontológicos Bauru SP Brasil Universidade de São Paulo, Faculdade de Odontologia de Bauru, Departamento de Dentística, Endodontia e Materiais Odontológicos, Bauru, SP, Brasil.; 5 Universidade Estadual Paulista Faculdade de Odontologia de Araraquara Departamento de Fisiologia e Patologia Araraquara SP Brasil Universidade Estadual Paulista - UNESP, Faculdade de Odontologia de Araraquara, Departamento de Fisiologia e Patologia, Araraquara, SP, Brasil.

**Keywords:** Lipopolysaccharides, Cell Culture Techniques, Dental Pulp, Pulpitis, Biomineralization

## Abstract

Simulating a bacterial-induced pulpitis environment in vitro may contribute to exploring mechanisms and bioactive molecules to counteract these adverse effects.

**Objective:**

To investigate the chronic exposure of human dental pulp cells (HDPCs) to lipopolysaccharides (LPS) aiming to establish a cell culture protocol to simulate the impaired odontogenic potential under pulpitis conditions.

**Methodology:**

HDPCs were isolated from four healthy molars of different donors and seeded in culture plates in a growth medium. After 24 h, the medium was changed to an odontogenic differentiation medium (DM) supplemented or not with E. coli LPS (0 - control, 0.1, 1, or 10 µg/mL) (n=8). The medium was renewed every two days for up to seven days, then replaced with LPS-free DM for up to 21 days. The activation of NF-κB and F-actin expression were assessed (immunofluorescence) after one and seven days. On day 7, cells were evaluated for both the gene expression (RT-qPCR) of odontogenic markers (*COL1A1, ALPL, DSPP*, and *DMP1*) and cytokines (*TNF, IL1B, IL8*, and *IL6*) and the production of reactive nitrogen (Griess) and oxygen species (Carboxy-H2DCFDA). Cell viability (alamarBlue) was evaluated weekly, and mineralization was assessed (Alizarin Red) at 14 and 21 days. Data were analyzed with ANOVA and post-hoc tests (α=5%).

**Results:**

After one and seven days of exposure to LPS, NF-κB was activated in a dose-dependent fashion. LPS at 1 and 10 µg/mL concentrations down-regulated the gene expression of odontogenic markers and up-regulated cytokines. LPS at 10 µg/mL increased both the production of reactive nitrogen and oxygen species. LPS decreased cell viability seven days after the end of exposure. LPS at 1 and 10 µg/mL decreased hDPCs mineralization in a dose-dependent fashion.

**Conclusion:**

The exposure to 10 µg/mL LPS for seven days creates an inflammatory environment that is able to impair by more than half the odontogenic potential of HDPCs *in vitro*, simulating a pulpitis-like condition.

## Introduction

Mesenchymal stem cells in dental pulp play a vital role in the defense and regenerative potential of the dentin-pulp complex against lesions by producing reparative dentinogenesis.^1,2^ However, the ability of dental pulp to recover from persistent infectious and inflammatory conditions is challenging. Clinically, inflamed pulp tissues are mostly characterized by spontaneous or long-lasting pain, and self-healing is not expected. Considering the anatomical restriction and limited circulation of the dental pulp, the inflammation process becomes self-destructive and irreversible.^2,3^

Histologically, teeth with irreversible pulpitis often present anaerobic gram-negative bacteria, which was not observed in normal/reversibly-inflamed pulps.^3^ Gram-negative bacteria outer membrane contains endotoxins known as lipopolysaccharides (LPS).^4^ LPS present potent biological effects, being able to stimulate pulp cells to produce reactive nitrogen/oxygen species,^5-8^ proteolytic enzymes (e.g., matrix metalloproteinases, especially MMP-9),^9-10^ and inflammatory cytokines (e.g., tumor necrosis factors – TNFs, and interleukins, especially IL-8, IL-6, and IL-1).^4,10-14^ The immunomodulatory effects of LPS on pulp cells are mainly mediated by cell receptors that activate the nuclear factor *kappa* B (NF-κB) signaling pathway.^4,8,14^ LPS are found in carious lesions of symptomatic and asymptomatic teeth at increasing numbers as the lesions get deeper and more painful.^15-17^ Therefore, the presence of LPS has been directly associated with pulpitis symptoms and its concentration seems to be positively correlated to the irreversibility of an inflammatory environment in dental pulp.^15-18^

Clinically, relatively low doses of stimuli in the early or resolving stages of bacterial invasion and caries development stimulate regenerative responses, inducing reactionary dentinogenesis.^2,19^ However, intense bacterial stimuli in active and chronic caries potentially impair regenerative processes.^19^ Therefore, many studies have investigated *in vitro* different LPS-challenged dental pulp cell populations, including dental pulp fibroblasts, odontoblasts, and dental pulp stem cells (DPSCs).^4-10,20-22^ Despite several studies on LPS effects in different pulp cells,^4^ there is not an established protocol to properly simulate the behavior of dental pulp cells under a bacterial-mediated degenerative pulpitis environment *in vitro*, i.e., able to impair biomineralization induced by pulp cells metabolism. There is a substantial divergence in the relationships between concentration and time of exposure to LPS with the inflammatory response and odontogenic potential of pulp cells *in vitro*.^4-10,20-22^ Therefore, depending on cell culture design, the regenerative potential of mesenchymal pulp cells may be stimulated or impaired, impacting the gene expression and protein translation of odontogenic markers, including type I collagen (partially encoded by *COL1A1*), alkaline phosphatase (encoded by *ALPL*), dentin sialophosphoprotein (encoded by *DSPP*), and dentin matrix acid phosphoprotein 1 (encoded by *DMP1*); all these events directly affect the ability of pulp cells to produce a mineralized matrix.^4,10,13,20-22^

Simulating an LPS-induced pulpitis environment *in vitro* may contribute exploring the mechanisms involved in pulp cell regeneration and screening bioactive molecules to counter these adverse effects.^23^ Thus, this study aimed to establish a cell culture protocol to simulate the impaired odontogenic potential of human dental pulp cells (HDPCs) under pulpitis conditions. The null hypotheses tested were that 1) the chronic exposure to LPS would not simulate *in vitro* the impaired odontogenic potential of pulp cells under pulpitis conditions, and 2) LPS concentrations would not differently modulate the inflammatory and odontogenic responses of HDPCs.

## Methodology

### Establishment and characterization of the primary culture of human dental pulp cells

Human dental pulp cells (HDPCs) were obtained from healthy erupted third molars (n=4) extracted from four different donors (two male and two female) aged 18 to 21 years (ethics approval under protocol no. 55269822.7.0000.5416, Local Research Ethics Committee). The pulp tissues were collected immediately after teeth extraction using a surgical hammer and endodontic files. Then, the tissues were enzymatically digested in individual tubes containing 3 mg collagenase type I (GIBCO, Invitrogen, Carlsbad, CA, USA) dissolved in 1 mL alpha minimum essential medium (α-MEM; supplemented with 100 IU/mL penicillin, 100 μg/mL streptomycin, 2 mmol/L glutamine, and 0.25 g/mL amphotericin B; all from GIBCO), for 3 h at 37°C and 5% CO_2_ in a humidified environment.^25^ Cells were cultured in a growth medium comprising α-MEM (GIBCO) supplemented with 10% fetal bovine serum (FBS, GIBCO) in 6-well plates (Kasvi, São José dos Pinhais, PR, Brazil) until reaching 80% confluency. Then, cells were subcultured by trypsinization (pool of the four teeth presented in passage #2) and used from passages #3 to #5 for the experiments.

The established cell culture was characterized by the presence of mesenchymal stem cell markers.^24^ Cells from passage #3 (n=2) were cultured until reaching 90% confluency. Then, cells were non-enzymatically dissociated (PBS-based dissociation buffer, GIBCO) and resuspended in tubes containing a staining buffer (2% bovine serum albumin in phosphate-buffered saline – PBS, Sigma-Aldrich, Saint Louis, MO, USA). Single-cell suspensions were transferred to individual tubes containing anti-human monoclonal antibodies conjugated with fluorescein isothiocyanate (FITC) or phycoerythrin (PE) against CD105, CD73, CD90, CD146, CD34, and CD45 (1:100, BD Biosciences, San Jose, CA, USA). After 45 min of incubation, cells were washed twice in PBS, resuspended in a new antibody-free staining buffer, and analyzed in a flow cytometer against FITC or PE controls (1×10^5^ events per sample, Accuri C6, BD Biosciences).

### Experimental design

The effects of LPS (*E. coli* O111:B4, Sigma-Aldrich) at different concentrations (0 - control, 0.1, 1, and 10 µg/mL) on HDPCs were tested under two different experimental conditions: #1) LPS concentrations diluted in FBS-free α-MEM (basal medium); and #2) LPS concentrations diluted in α-MEM + 10% FBS (growth medium). HDPCs (5×10^3^ cells) were seeded in 96-well plates (TPP, Trasadingen, SH, Switzerland) in a growth medium. After 24 h, the cell culture medium was changed (100 µL) by the treatments under the two conditions, as previously described, and renewed every two days. After seven days, cell viability was evaluated as described. Next, all media were renewed by the same LPS-free odontogenic differentiation induction medium comprising a growth medium supplemented with 5 mM β-glycerophosphate (Santa Cruz Biotechnology, Santa Cruz, CA, USA) and 50 μg/mL ascorbic acid (Fisher Chemical, Fair Lawn, NJ, USA) for further 14 days, totaling 21 days of culture. At this time point (21 days), cells were evaluated for viability and mineralized matrix formation, as described below.

**Figure 1:**
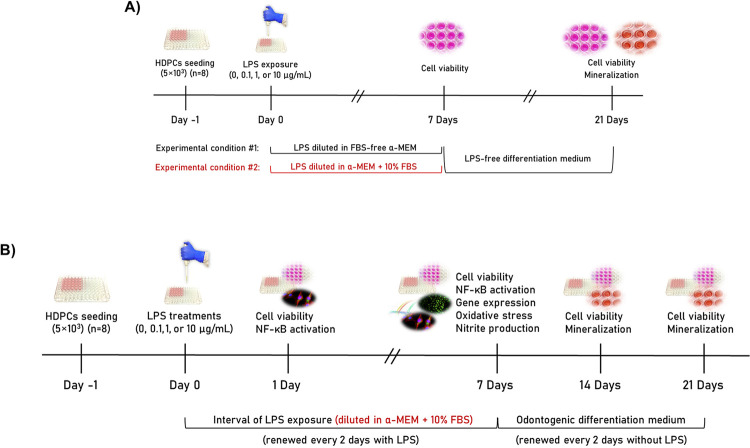
A) Schematic representation of the experimental design to assess the effects of different concentrations of lipopolysaccharides (LPS) on human dental pulp cells (HDPCs). Cells were tested under two different experimental conditions: #1) LPS concentrations diluted in FBS-free α-MEM (basal medium); or #2) LPS concentrations diluted in α-MEM + 10% FBS (growth medium). After 24 h of cell seeding (Day 0), the culture media were supplemented or not with LPS (0 - control, 0.1, 1, and 10 µg/mL) for seven days. After this time interval, cell viability was evaluated, and cells were cultured in an odontogenic differentiation medium without LPS for further 21 days, when cell viability and mineralized matrix formation were evaluated. B) Schematic representation of the second experimental design. After 24 h of cell seeding (Day 0), the culture medium was supplemented or not with LPS (0 - control, 0.1, 1, and 10 µg/mL) for seven days. After this time interval, cells were cultured in an odontogenic differentiation medium without LPS for up to 21 days. Cell viability was assessed weekly (from Day 1). On day 7, cells were also evaluated for NF-κB activation, gene expression, oxidative stress, and nitrite production. The formation of a mineralized matrix was evaluated after 14 and 21 days

The experimental condition #2 showed a dose-dependent effect of LPS on the mineralization ability of HDPCs. Therefore, this protocol was set for further experiments. The response of HDPCs to chronic exposure to LPS (*E. coli* O111:B4, Sigma-Aldrich) concentrations (0 - control, 0.1, 1, and 10 µg/mL) diluted in an odontogenic condition was assessed. Therefore, HDPCs (5×10^3^) were seeded in 96-well plates (TPP) in a growth medium (Day -1). After 24 h (Day 0), cells were exposed to the treatments. After further 24 h (Day 1), cell viability was evaluated, and the medium was replaced (100 µL) with the treatments (culture medium supplemented or not with LPS concentrations) and renewed every two days, totaling four changes. This culture medium was supplemented for odontogenic differentiation as previously described. On day 7, cells were evaluated for viability, gene expression of odontogenic markers and cytokines, reactive nitrogen species (nitrite) production, and reactive oxygen species (general oxidative stress) production. After the seventh day, the media were renewed with the same LPS-free odontogenic medium for further 14 days, totaling 21 days of culture. At 14 and 21 days, cells were evaluated for viability and mineralized matrix formation. The activation of the nuclear transcription factor kappa B (NF-κB) and F-actin expression were investigated after 1 or 7 days of LPS exposure. Figure 1 shows the schematic representation of the experimental designs of this study, and the experimental protocols are detailed below.

### Cell viability (n=8)

Cells were incubated with a 10% alamarBlue solution (Invitrogen, Carlsbad, CA, USA) in a serum-free medium at 37°C and 5% CO_2_. After 3 h, an aliquot of the supernatant was collected to measure fluorescence intensity at 560 nm excitation and 590 nm emission (Synergy H1, BioTek, Winooski, VT, USA).^25^ Cells cultured in an LPS-free medium on day 1 were set as 100% viability.^25^ Then, cells were washed in PBS and cultured until the next time intervals (paired samples).

### Mineralized matrix formation (n=8)

Cells were fixed with 70% ethanol at 4°C, rinsed with deionized water, and stained with 40 mM of Alizarin Red S solution (pH 4.2, Sigma-Aldrich) for 15 min under shaking.^25^ Then, the background staining was removed by rinsing twice with deionized water. After drying, a mineralized matrix was visualized using a light microscope (Olympus, Tokyo, Japan). The calcium deposits were solubilized in a cetylpyridinium chloride solution (10 mM, pH 7.0; Sigma-Aldrich) to allow measuring the absorbance at 570 nm (Synergy H1).^25^ Cells cultured in an LPS-free odontogenic differentiation medium represented 100% of mineralization.

### Gene expression of odontogenic markers and cytokines (n=6)

After 3 h since the last exposure to LPS (Day 7), total RNA was extracted using the TRIzol reagent (Invitrogen) protocol followed by treatment with DNAse (Sigma-Aldrich). Then, 500 µg of purified RNA was used as the template for first-strand complementary DNA synthesis using the High-Capacity cDNA Reverse Transcription kit (Applied Biosystems, Life Technologies, Foster City, CA, USA). Real-time polymerase chain reactions were conducted using TaqMan gene expression assays (Applied Biosystems) to amplify *COL1A1* (Hs01076756_g1), *ALPL* (Hs01029144_m1), *DSPP* (Hs00171962_m1), *DMP1* (Hs01009391_g1), *TNF* (Hs00174128_m1), *IL1B* (Hs01555410_m1), *IL8* (Hs_00174103_m1), and *IL6* (Hs00174131_m1) sequences. Reactions followed the TaqMan Fast Advanced Master Mix (Applied Biosystems) conditions processed in the StepOnePlus system (Applied Biosystems). The relative expression (fold change) for each gene was calculated using 2^-^ equations with *GAPDH* (Hs02786624_g1) as the reference gene.^25^

### Reactive nitrogen species production (n=8)

Additionally, also 3 h after the last exposure to LPS (Day 7), the culture medium in contact with the cells was mixed with a modified Griess reagent (1:1, Sigma-Aldrich) in compartments of a 96-well plate. After 10 min of incubation in the dark, the absorbance of the resulting reaction was determined at 540 nm (Synergy H1).^5,6^ A cell-free culture medium mixed with Griess reagent (1:1) was used as a blank and the data were presented in fold increase of control (LPS-free medium).

### Reactive oxygen species production (n=8)

Furthermore, 3 h after the last exposure to LPS (Day 7), the culture medium in contact with the cells was replaced with 100 μL of a solution containing a fluorescent probe for intracellular oxidative stress (Carboxy-H2DCFDA, Invitrogen, 10 μM in PBS) for 15 min. After incubation, the cells were washed twice with PBS and fluorescence intensity was determined at 492 nm excitation and 517 nm emission (Synergy H1).^5,6^ The data were presented in fold increase of control (LPS-free medium). The same protocol was used for the qualitative evaluation, and samples (n=2) were analyzed at 10× magnification with fluorescence microscopy (Leica, Austin, TX, USA).

### NF-κB activation and F-actin expression (n=4)

In the period of 1 h after exposure to LPS concentrations on days 1 and 7, cells were fixed with 4% formaldehyde (Sigma-Aldrich) at 4°C for 10 min, washed twice in PBS, and permeabilized with 0.1% Triton-X 100 (Sigma-Aldrich) for 5 min. Then, cells were treated with 5% BSA (Sigma-Aldrich) for 30 min to block non-specific bindings, and then were incubated with monoclonal mouse anti-human NF-κB subunit p65 primary antibody (1:100, Santa Cruz Biotechnology, Santa Cruz, CA, USA) overnight at 4°C. Then, cells were incubated with goat anti-mouse IgG-FITC-conjugated secondary antibody (1:100, Jackson Immunoresearch Laboratories, West Grove, PA, USA) for 1 h. Additionally, the actin filaments (F-actin) were stained with a rhodamine phalloidin probe (1:20, ActinRed555 ReadyProbes reagent, Invitrogen), followed by incubation with Hoechst 33342 (1:5000, Invitrogen) for 15 min to counterstain cell nuclei.^25^ Cells were analyzed at 40× magnification in a fluorescence microscope (Leica).

### Data analyses

All analyses considered the pre-established α=5%. The experiments were performed in two experimental replicates to allow verifying the reproducibility of results. The number of biological replicates of each experiment was calculated with the G*Power software (version 3.1, University Dusseldorf, Dusseldorf, NW, Germany) to reach at least 80% power. The data were analyzed with one-way ANOVA and repeated-measures ANOVA. Multiple comparisons were calculated with Tukey, Sidak, or Games-Howell post-hoc tests, according to the assumptions of normality (Shapiro-Wilk) and equality of variances (Levene). These analyses were performed with the SPSS (version 26.0, IBM Inc., Chicago, IL, USA) and GraphPad Prism (version 9.0, GraphPad, San Diego, CA, USA) software.

## Results

**Figure 2:**
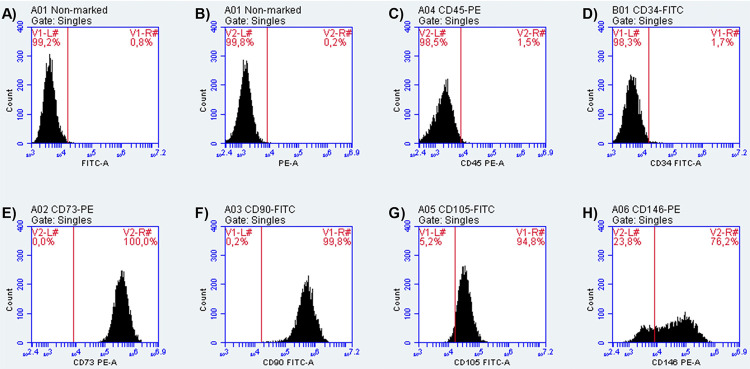
Flow cytometry histograms of the mesenchymal stem cell antigens expressed in the established primary culture of human dental pulp cells (HDPCs). Sections A) and B) show representative negativity of FITC and PE channels of fluorescence controls, respectively. Representative histograms and percentage of expression are given for C) CD45, D) CD34 (both negative markers), E) CD73, F) CD90, G) CD 105, and H) CD146 (all positive markers)

Figure 2 shows the immunophenotypic characterization by flow cytometry of the primary HDPCs culture. Most cells (≥76.2% cell population) expressed positive mesenchymal stem cell markers (CD73, CD90, CD105, and CD146), whereas negative markers (CD45 and CD34) were minimally expressed (≤1.7% cell population). Therefore, the established HDPCs culture was mostly composed of mesenchymal stem cells.

**Figure 3:**
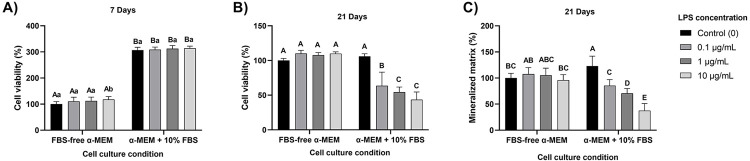
A) Cell viability after seven days (% of control FBS-free medium) for both experimental conditions: #1) LPS concentrations diluted in FBS-free α-MEM (basal medium); or #2) LPS concentrations diluted in α-MEM + 10% FBS (growth medium). Columns are means and error bars are standard deviations (n=8). Uppercase letters compare the different conditions and lowercase letters compare the LPS concentrations within the same experimental condition. Distinct letters are statistically different (two-way ANOVA/Sidak, α=5%). B) Cell viability after 21 days (% of control FBS-free medium) for both experimental conditions. Columns are means and error bars are standard deviations (n=8). Distinct letters compare each column, showing statistical differences (two-way ANOVA/Sidak, α=5%). C) Mineralized matrix formation after 21 days (% of control FBS-free medium) for both experimental conditions. Columns are means and error bars are standard deviations (n=8). Distinct letters compare each column, showing statistical differences (two-way ANOVA/Sidak, α=5%)

Cells cultured with serum presented around 3-fold higher viability after seven days (Figure 3A), indicating a higher proliferation rate under serum supplementation. This shorter period did not show an interaction effect for treatments and culture conditions regarding cell viability (p=0.62), and only 10 μg/mL LPS increased cell viability without FBS compared to control (p=0.02, Figure 3A). Conversely, there were no differences in cell viability for neither LPS concentrations diluted in a medium containing FBS (p≥0.75, Figure 3A). Overall, cell viability was lower when cells were cultured without FBS after seven days (p<0.0001, Figure 3A). At 21 days, there was an interaction effect for cell viability and formation of a mineralized matrix under the two conditions and LPS concentrations (p<0.0001). The absence of LPS (control) did not show significant differences in cell viability whether FBS was added to the culture medium or not. However, a detrimental effect of LPS on HDPCs metabolism was detected only when FBS was added to the culture medium (Figure 3A). In that experimental condition, all LPS concentrations significantly reduced cell viability compared to the control (p<0.0001, Figure 3B). The same phenomenon was seen in the formation of a mineralized matrix. The different concentrations of LPS exerted a detrimental effect only in the presence of FBS in the culture medium. That detrimental effect was concentration-dependent and always statistically significant compared to the control (p<0.0001, Figure 3C). However, no LPS concentrations affected the formation of a mineralized matrix when cells were cultured under a basal medium (p≥0.99, Figure 3C). Considering that the dose-dependent pattern of LPS decreased the mineralization ability of HDPCs without affecting cell viability at day 7 under the experimental condition #2, this protocol was chosen for further experiments.

**Figure 4:**
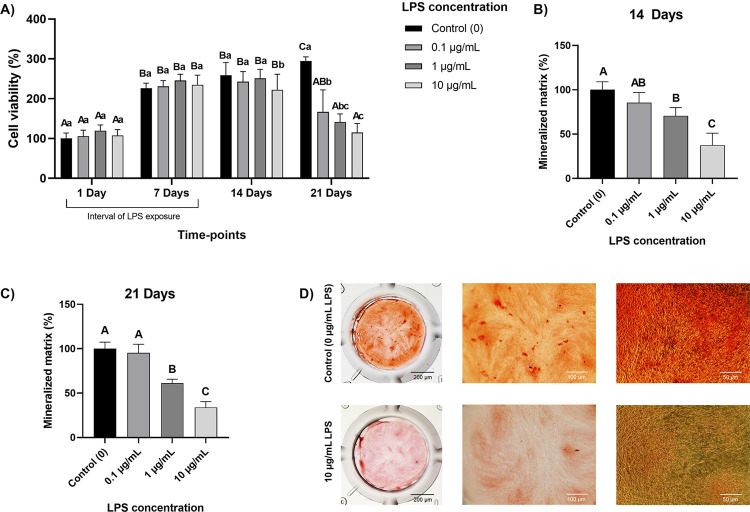
A) Cell viability (% of 1-day control) of human dental pulp cells (HDPCs) cultured with the LPS concentrations (0 - control, 0.1, 1, and 10 µg/mL) diluted in α-MEM + 10% FBS assessed weekly (on days 1, 7, 14, and 21). Columns are means and error bars are standard deviations (n=8). Uppercase letters compare each LPS concentration at different time points and lowercase letters compare LPS concentrations within the same time point. Distinct letters are statistically different (repeated-measures ANOVA/Sidak, α=5%). B) Mineralized matrix formation (% of control) by HDPCs cultured with the LPS concentrations after 14 days. Columns are means and error bars are standard deviations (n=8). Distinct letters indicate statistically different groups (one-way ANOVA/Tukey, α=5%). C) Mineralized matrix formation (% of control) by HDPCs cultured with 0 or 10 µg/mL LPS after 21 days. Columns are means and error bars are standard deviations (n=8). Groups indicated with distinct letters are statistically different (t-test, α=5%). D) Representative images of mineral deposits in an extracellular matrix formed by HDPCs cultured with 0 or 10 µg/mL LPS after 21 days (in the row: an overview of a 96-well plate compartment stained with Alizarin Red, detailed view of each well, and inverted light microscopy image)

Cell viability was not modulated by any LPS concentration during the exposure interval (one or seven days, p≥0.52, Figure 4A). Seven days after the end of exposure (14 days of culture), only 10 µg/mL decreased cell viability compared to the control of the same period (p=0.02, Figure 4A). Fourteen days after the end of exposure (21 days of culture), all LPS concentrations decreased cell viability compared to the control of the respective period (p<0.0001, Figure 4A). The formation of a mineralized matrix decreased in a dose-dependent manner seven days after the end of exposure (14 days of culture). The reduction was significant for 1 µg/mL and 10 µg/mL concentrations (p<0.0001), reaching 62.5% for the highest concentration (Figure 4B). Fourteen days after the end of exposure (21 days of culture), 1 and 10 µg/mL LPS significantly reduced (p<0.0001) mineralized matrix formation by HDPCs by 39 and 66%, respectively (Figure 4C).

**Figure 5:**
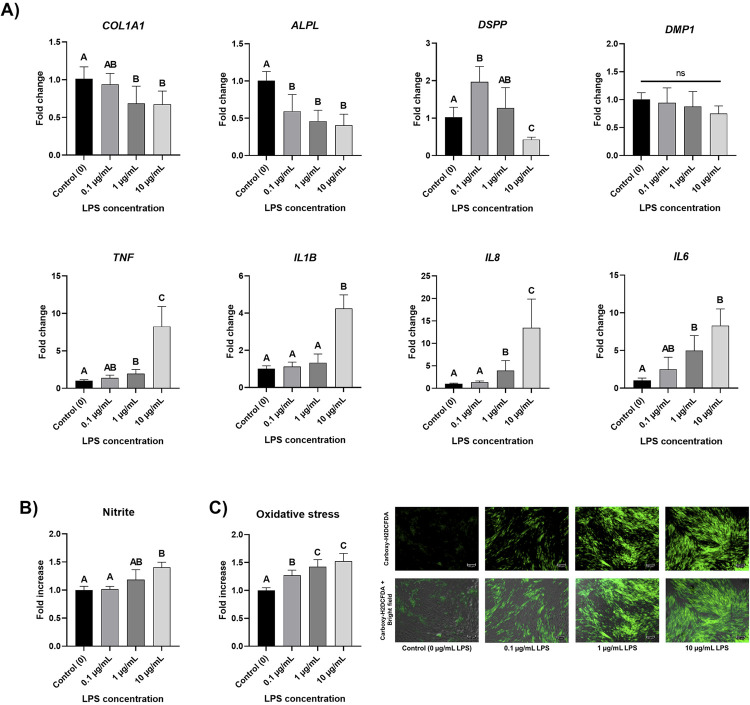
A) Gene expression modulation (fold change of control) of odontogenic markers (ALPL and DSPP) and cytokines (TNF, IL1B, and IL8) by human dental pulp cells (HDPCs) cultured with LPS concentrations (0 - control, 0.1, 1, and 10 µg/mL) on day 7. Columns are means and error bars are standard deviations (n=6). Distinct letters indicate that groups are statistically different (one-way ANOVA/Tukey or Welch’s one-way ANOVA/Games-Howell, α=5%). B) Reactive nitrogen species (nitrite, fold increase of control) production by human dental pulp cells (HDPCs) cultured with LPS concentrations (0 - control, 0.1, 1, and 10 µg/mL) on day 7. Distinct letters are statistically different (Welch’s one-way ANOVA/Games-Howell, α=5%). C) Reactive oxygen species (oxidative stress, fold increase of control) production by human dental pulp cells (HDPCs) cultured with LPS concentrations (0 - control, 0.1, 1, and 10 µg/mL) on day 7. Distinct letters are statistically different (one-way ANOVA/Tukey, α=5%). On the right, there are representative images (10×) of intracellular oxidative stress (green fluorescence – Carboxy-H2DCFDA probe) in human dental pulp cells (HDPCs) cultured with LPS concentrations (0 - control, 0,1, 1, and 10 µg/mL) on day 7. In the bottom row of the images, the same green fluorescence images (with higher brightness and contrast) merge with the bright field, showing cell confluency

Figure 5A shows the modulation of the gene expression of odontogenic markers (*COL1A1, ALPL, DSPP*, and *DMP1*) and cytokines (*TNF, IL1B, IL8*, and *IL6*) after seven days of exposure to LPS. Only 1 and 10 µg/mL LPS down-regulated *COL1A1* gene expression (p≤0.026, Figure 5A). All LPS concentrations down-regulated the gene expression of *ALPL* (p≤0.01, Figure 5A). The *DSPP* gene was up-regulated by 0.1 µg/mL LPS (p=0.005, Figure 5A) and down-regulated by 10 µg/mL LPS by half (p=0.009, Figure 5A). No concentration modulated *DMP1* gene expression (p=0.052, Figure 5A). Overall, 1 and 10 µg/mL LPS significantly up-regulated inflammatory-related gene expression. *TNF, IL1B, IL8*, and *IL6* genes were significantly up-regulated around 8-fold, 4-fold, 13-fold, and 8-fold by 10 µg/mL LPS, respectively (p≤0.03, Figure 5A). After seven days of exposure to LPS, only 10 µg/mL LPS significantly increased nitrite production (p<0.0001, Figure 5B). However, all LPS concentrations increased oxidative stress in a dose-dependent manner (p<0.0001, Figure 5C), increasing around 1.5× for 10 µg/mL LPS.

**Figure 6:**
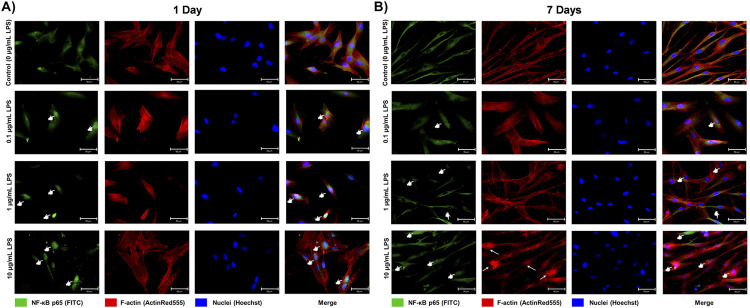
Immunofluorescence of NF-κB subunit p65 (green fluorescence – FITC) and actin filaments (F-actin, red fluorescence – ActinRed555) expressed in human dental pulp cells (HDPCs) after one and seven days of exposure to 0 (control) or 10 µg/mL LPS. The cell nuclei (blue fluorescence – Hoechst) were counterstained and the channels were merged (40×). After one and seven days, cell cultures without LPS (control) showed an expression of p65 mostly in the cytoplasm. Conversely, the exposure to 10 µg/mL LPS increased the nuclear translocation of p65 (pointers), demonstrating the activation of the NF-κB transcription factor. Chronic LPS exposure also abnormally affected the F-actin distribution around the nuclei after seven days (arrows)

After one and seven days, the nuclear translocation of NF-κB subunit p65 increased in a dose-dependent fashion for the LPS concentrations, showing the activation of the NF-κB transcription factor (Figure 6). Additionally, after seven days, the exposure to 10 µg/mL LPS demonstrated more intense effects on NF-κB, also affecting the F-actin distribution, aggregated around the nucleus (Figure 6).

## Discussion

Research methods and experimental models to study pulpitis have been critically highlighted, urging for the development of models to simulate the clinical scenario of prolonged bacterial stimulation and progression of caries towards the pulp.^23^ Thus, this study investigated chronic exposure to LPS as an *in vitro* model to simulate the regenerative potential of pulp cells under pulpitis conditions. Exposing HDPCs to the concentrations of 1 and 10 µg/mL LPS for seven days (chronically) properly up-regulated the inflammatory response and impaired the odontogenic-related regenerative potential. Therefore, the first hypothesis of this study was accepted, whereas the second was rejected.

Although not involved in the caries process, *E. coli* LPS have been the most used source of endotoxin due to reliable and reproducible results.^4,23^ LPS elicit a crosstalk effect among different signaling pathways in pulp cells, differing from the stimulation with isolated cytokines, which may impact reciprocal molecular mechanisms involved in the immunomodulation and regenerative potential of DPSCs.^4^ The LPS concentrations were selected based on previous *in vitro* studies stimulating HDPCs,^10,13,14,21^ and considered a range within the quantification of endotoxins *in vivo* in symptomatic teeth, reaching around 22 EU/ml,100 EU/mL, or 4.4 µg/mL.^26^ Considering the high population of DPSCs in the established HDPCs cell culture, as demonstrated by flow cytometry, the protocol used in this study was a convenient model to modulate the pro- and anti-inflammatory capacities of non-immune pulp cells stimulated by gram-negative bacteria by-products, as it occurs clinically. The DPSCs’ ability to modulate inflammation and induce mineralization clinically relates with the reparative tertiary dentinogenesis, where lost odontoblasts are replaced by mesenchymal stem cells differentiated into odontoblast-like cells.^1,2^

First, the effects of LPS on HDPCs were tested under serum-free or supplemented conditions. Cells cultured with serum presented higher viability, indicating a higher proliferation rate under serum supplementation. This result was expected since FBS provides many proteins, hormones, nutrients, and growth factors that facilitate cell proliferation. LPS affected cell viability only when cells were grown with serum, indicating that cells proliferating under LPS stimuli resulted in the confluency of LPS-sensitized cells. Conversely, when cultured without serum, cells probably reached confluency after the end of LPS stimuli (i.e., after the first week), thus recovering and generating new cells that had never been in direct contact with endotoxins. The presence of serum is a critical factor in LPS sensitization since it provides soluble CD14 protein, which is a pivotal coreceptor for TLR-4 activation,^27^ especially considering that DPSCs (as MSCs) do not endogenously produce this protein.^24^ Therefore, considering a clinical translation in which mesenchymal stem cells proliferate under LPS stimuli,^26,28^ the FBS-supplemented experimental condition was set for further experiments.

HDPCs were stimulated with different LPS concentrations every two days for up to a week. During the exposure interval (seven days), no LPS concentrations modulated cell viability in this study. Consistently, the exposure to LPS (in a range of 0.01 to 10 µg/mL) for a week was the most prolonged time interval to evoke an immunomodulatory profile without affecting cell viability in other studies.^10,13,22^ This concentration interval presented little effect on the apoptosis of human DPSCs,^22^ supporting our findings. In this study, 10 µg/mL LPS decreased cell viability after 14 days of culture, even seven days after the end of exposure. After 21 days of culture, all LPS concentrations decreased cell viability, especially the higher concentrations. These results may relate to a stress-induced senescence phenotype elicited by repeated LPS stimulation (more than three times), justifying seven days chosen for chronic LPS stimulation.^29,30^ Senescent mesenchymal stem cells morphologically present a flattened cell structure with an abnormal distribution of actin filaments (F-actin) around the nucleus,^30^ indicating the impairment of proper globular actin polymerization into fibrous actin. This study noted these events after a week of exposure to LPS, thus supporting this hypothesis. Since F-actin is directly involved in cell structure and motility, these events also suggest that chronically LPS-challenged cells may have their adhesive and migration abilities impaired, clinically reflecting poor regenerative outcomes. These events should be investigated in further studies.

General oxidative stress and nitrite production were used to measure the production of reactive oxygen and nitrogen species (RONS) by HDPCs.^5,6^ These free radicals are physiologically self-regulated, but their excessive formation is a marker of the progression of an inflammation process, resulting in chronic inflammation.^7^ Overall, LPS exposure exerted a dose-dependent response on RONS production by HDPCs after seven days of stimulation. Previous studies have demonstrated that LPS up-regulated this production,^5-8^ and the repeated LPS stimulation contributed to increasing senescence and DNA damage in DPSCs,^30^ indicating a response similar to a chronic inflammation process. Moreover, increased RNS concentrations (e.g., nitric oxide) directly mediate the up-regulation of the gene expression and synthesis of interleukin (IL)-8 in HDPCs.^8^ Accordingly, the exposure to 1 and 10 µg/mL LPS remarkably up-regulated *TNF, IL6*, and, especially, *IL8* gene expression in this study. The up-regulation of these pro-inflammatory-related genes by HDPCs induced by LPS corroborates other studies.^10,12-14^ The TNF and IL cytokine families are potent chemoattractants that mediate the recruitment and activation of defense cells intensely expressed in symptomatic teeth diagnosed with irreversible pulpitis, especially IL-8,^11,31,32^ thus validating the protocol suggested in this study.

The immunomodulatory effects of LPS on pulp cells are mediated by specific toll-like receptors (-2 and -4) that activate NF-κB signaling usually by the myeloid differentiation factor 88 (MyD88) pathway.^29,30,33^ The NF-κB protein complex is a critical transcription factor that orchestrates inﬂammatory response by regulating gene expression and cytokine synthesis.^34^ The NF-κB p65 subunit presents transactivation domains pivotal for NF-κB transcriptional activity, characterized by its translocation from the cytoplasm (where it concentrates under normal physiological conditions) into the nucleus, binding to promoter regions of target genes and regulating their expression.^34^ The involvement of NF-κB in the RNS-induced IL-8 expression by HDPCs has been previously demonstrated.^8^ In our study, the immunofluorescence images showed that the proposed LPS stimulation protocol activated the NF-κB transcription factor, leading to the up-regulation of cytokines gene expression and RONS production. These events indicate the hallmark involvement of the NF-κB signaling pathway in the LPS-mediated immunomodulatory effects on HDPCs.

The active cellular NF-κB pathway in DPSCs from traumatically exposed pulps can down-regulate their odontogenic potential (*Dspp*/DSP gene/protein expression).^35^ These events agree with our results, especially for the 1 and 10 µg/mL LPS concentrations, which sharply down-regulated *COL1A1, ALPL*, and *DSPP* gene expression levels and decreased mineralized matrix formation by HDPCs. Other researchers found similar effects,^10,20,22^ which may be partially related to a persistent NF-κB activation that could impair the osteogenic differentiation of mesenchymal stem cells by competing with the Wnt/β-catenin signaling involved in tertiary dentinogenesis.^36-38^ However, the absent modulation of *DMP1* gene expression should be considered and attributed to the cell transcriptional activity at the specific time point used for analysis, thus we suggest that evaluating other time points should be regarded in further studies. Moreover, one may consider that high cytokine levels induced by LPS may up-regulate the expression and synthesis of matrix metalloproteinases (MMPs) including -1, -2, -3, and -9;^10,39^ these enzymes mediate collagen degradation, thus impairing the proper maturation of the mineralized matrix. Considering that the early stages of bacterial invasion and caries development stimulate reactionary dentinogenesis,^2,19^ the low dosage of 0.1 µg/mL LPS may have increased the odontogenic potential of HDPCs, corroborating other *in vitro* studies.^13,20-22^ This indicates that relatively high LPS concentrations are required to evoke degenerative responses *in vitro*.

We highlight that, in this study, the kinetics of LPS stimulation impaired the odontogenic-related regenerative potential of HDPCs even after interrupting the endotoxin stimulus, indicating the promotion of a chronic inflammatory effect, and creating an *in vitro* pulpitis-like environment. However, this two-dimensional experimental model is quite far from accurately simulating the complex dynamic pulp inflammatory response as bacterial contamination progresses. Apart from the intrinsic limitations of cell culture studies (e.g., batch-to-batch variation of serum, changes in cell morphology, metabolism, and the limited interactions with extracellular components), the crosstalk effect between different signaling pathways within and between DPSCs, fibroblast, endothelial, and defense cells explains the immunomodulation and regenerative potential under pulpitis conditions.^4,23^ However, further research shall benefit from the model proposed in this study to find biomarkers of pathogen-mediated pulpitis and explore molecules and biomaterials to minimize or hinder the down-regulation of odontogenic differentiation of HDPCs after chronic exposure to LPS. Although both 1 and 10 µL/mL LPS exerted significant effects, using the higher concentration (10 µL/mL) shall provide a higher challenge, thus providing safer responses. Since regenerative endodontics is an emerging field, researchers should consider balancing inflammation and the degenerative effects of chronic LPS stimuli in bacteria-affected environments to properly stimulate an osteo/odontoblastic phenotype to induce mineralization by resident stem cells, thus resulting in tissue regeneration. This should help predict better clinical outcomes of minimally invasive vital pulp therapies under pulpitis conditions.

## Conclusion

The exposure to 10 µg/mL LPS for seven days creates an inflammatory environment able to impair by more than half the odontogenic potential of HDPCs *in vitro*. This protocol may be suitable to simulate the regenerative potential of pulp cells under pulpitis conditions *in vitro*.
